# Gender Influences on Depression Severity in Patients With Urinary Incontinence

**DOI:** 10.7759/cureus.82477

**Published:** 2025-04-17

**Authors:** Marc Ganz, Marco Halabi, Myles Goliger, David Sezanayev, Benjamin Nasimov, Samson Balakhani, Nekhama Riznyk, Michael West

**Affiliations:** 1 Urology, State University of New York Downstate Health Sciences University, Brooklyn, USA; 2 Urology, Touro College of Osteopathic Medicine, New York City, USA; 3 Internal Medicine, New York Institute of Technology College of Osteopathic Medicine, Old Westbury, USA; 4 Dentistry, Touro College of Dental Medicine, Hawthorne, USA; 5 Internal Medicine, State University of New York Downstate Health Sciences University, Brooklyn, USA; 6 Department of Urology, Maimonides Medical Center, Brooklyn, USA

**Keywords:** depression, gender-based differences, lower urinary tract symptoms (luts), quality of life, urinary incontinence (ui)

## Abstract

Introduction

Urinary incontinence is a prevalent lower urinary tract symptom (LUTS) that significantly impacts quality of life and poses a substantial healthcare burden. Concurrently, depression remains a major public health concern, contributing to disability and diminished well-being. While prior studies have documented a strong association between LUTS and depression, the extent to which gender differences modify this relationship remains unclear. This study investigates whether female patients with LUTS exhibit higher depression scores than their male counterparts, adjusting for demographic factors.

Methods

Utilizing data from the National Health and Nutrition Examination Survey (NHANES) (August 2021-August 2023), we analyzed the association between LUTS and depression among 2,326 individuals. LUTS was defined based on self-reported urinary leakage, while depression severity was assessed using the Patient Health Questionnaire-9 (PHQ-9). A linear regression model was employed to examine gender differences in depression scores, controlling for age, race, education, and marital status.

Results

The study cohort had a mean age of 59.47 years and was predominantly female (68.9%). Gender was not significantly associated with depression severity among LUTS patients (β = 0.252, 95% CI: -0.192 to 0.697, p = 0.266). However, age was a significant predictor, with each additional year correlating with a 0.066-point decrease in depression score (p < 0.001). Educational attainment and marital status also influenced depression severity, with college graduates exhibiting significantly lower depression scores (β = -2.267, p < 0.001) and unmarried individuals reporting higher scores (β = 1.411, p < 0.001).

Conclusion

While depression is generally more prevalent in women, our findings suggest that the presence of LUTS does not significantly modify this gender disparity. Instead, factors such as age, education, and marital status appear to exert a more substantial influence on depression severity in this population. These results underscore the importance of addressing broader sociodemographic determinants when assessing mental health outcomes among LUTS patients. Future research should explore whether specific LUTS subtypes or symptom severity differentially impact mental health in men and women.

## Introduction

Urinary incontinence is a common condition affecting millions of adults worldwide, with estimates suggesting that between 20% and 40% of the adult population experiences some form of urinary dysfunction, such as frequency, urgency, or incontinence [1]. These symptoms not only diminish quality of life but also impose a significant burden on healthcare systems globally [2]. Among the various manifestations of LUTS, urinary incontinence - defined as the involuntary loss of urine - is one of the most distressing and socially impactful symptoms. In parallel, depression is a major public health concern that affects over 250 million people worldwide and is recognized by the World Health Organization as a leading cause of disability [3]. Depression often results in considerable personal, social, and economic costs [4].

A growing body of literature has documented a strong association between LUTS and depression, suggesting that the distress caused by urinary symptoms may contribute to the development or exacerbation of depressive symptoms [5-7]. Studies have shown that individuals with LUTS are more likely to experience depression, potentially due to factors such as social embarrassment, diminished self-esteem, and chronic stress responses [8].

It is well established that depression is more prevalent in women than in men, a disparity attributed to various biological, hormonal, and psychosocial factors [9,10]. Moreover, LUTS - especially urinary incontinence - is often more common among women, potentially due to anatomical differences, the impact of childbirth, and other gender-specific factors [11,12]. This increased prevalence of LUTS in women may further exacerbate depressive symptoms by imposing additional psychosocial stressors, such as social stigma and challenges in daily functioning. Consequently, the coexistence of LUTS and depression might have a compounded effect on women compared to men [13].

Beyond gender, other demographic factors, including race, educational attainment, and marital status, have been shown to influence both the prevalence of LUTS [14,15] and the severity of depressive symptoms [16]. These variables serve as important confounders and mediators in this relationship, highlighting the need for analyses that control for these factors when assessing how LUTS may differentially modify depression in men versus women.

This study aims to utilize data from the National Health and Nutrition Examination Survey (NHANES) to investigate how depression manifests among patients with LUTS, specifically urinary incontinence, with a specific focus on potential gender differences. By adjusting for age, race, education, and marital status, our analysis seeks to determine whether female patients with LUTS exhibit higher depression scores than their male counterparts and to explore how these associations are influenced by demographic factors.

## Materials and methods

Data collection

Our study analyzed data from the NHANES, a project administered by the CDC [16]. We utilized data collected from the August 2021-August 2023 NHANES questionnaire. The analysis focused on three core sections within the NHANES dataset: Demographics, Urology, and Depression. The Urology section provided information on urinary leakage, which we used to construct a binary variable indicating the presence of LUTS. The Depression section contained the nine items of the Patient Health Questionnaire (PHQ-9), which were used to derive a composite depression score. This design allowed us to examine the relationship between LUTS and depression in a nationally representative sample. The study protocol was approved by the Physician's Journal of Medicine, based in Queens, New York, USA, under the approval number F2025034.

Variables

Participants were included in the analysis if they exhibited LUTS. The LUTS variable was derived from responses to the question on urinary leakage; a response of “Never” was coded as 0 (indicating no LUTS), while any other valid response (e.g., “less than once a month,” “a few times a month,” “a few times a week,” or “every day and/or night”) was coded as 1 (indicating the presence of LUTS). The primary dependent variable was the composite depression score. This score was calculated by summing the responses from the nine PHQ-9 items, with each item scored on a scale from 0 (“not at all”) to 3 (“nearly every day”), yielding a total score ranging from 0 to 27. Standard clinical thresholds were applied to categorize depression severity as follows: minimal (0-4), mild (5-9), moderate (10-14), moderately severe (15-19), and severe (20-27). To control for potential confounding factors, age, race, education, and marital status variables were included as covariates in the analysis:

Statistical analysis

All statistical analyses were performed using R version 4.2.0 (R Foundation for Statistical Computing, Vienna, Austria). Descriptive statistics were computed to summarize the demographic and health characteristics of the LUTS subgroup, including means, standard deviations, and ranges for continuous variables, as well as frequencies with percentages for categorical variables. A linear regression model was then fitted to assess whether there were differences in depression scores between male and female patients with LUTS while adjusting for age, race, education, and marital status. In this model, the composite depression score served as a continuous outcome variable, and the beta coefficients represent the expected change in the depression score for a one-unit change in the predictor (or the difference between groups for categorical variables). The significance of associations was determined at a two-sided p-value threshold of 0.05, with 95% confidence intervals calculated for all estimates.

## Results

A total of 2,326 patients with LUTS were included in the analysis. The average age was 59.47 years. The sample was predominantly female, with 1,602 females (68.9%) compared to 724 males (31.1%). In terms of race, 68.4% of participants were White, 12.9% were Hispanic, and 9.6% were Black. Educational attainment varied, with 3.2% reporting less than a ninth-grade education, 7.0% completing ninth to 11th grade, 19.3% graduating high school or obtaining a General Educational Development (GED), 32.9% having some college or an Associate of Arts (AA) degree, and 37.5% being college graduates or above. Marital status was nearly evenly split, as 54.5% of participants were married, and 45.4% were not married. In addition, based on PHQ-9 scores, 60.6% of patients had minimal depression (score: 0-4), 22.6% had mild depression (score: 5-9), 10.0% had moderate depression (score: 10-14), 4.2% had moderately severe depression (score: 15-19), and 1.6% had severe depression (score 20-27) (Table 1).

**Table 1:** Demographic and clinical characteristics of patients with LUTS LUTS, lower urinary tract symptom

Variable	Male LUTS n (%)	Female LUTS n (%)	Total n (%)
Count	724 (31.1)	1602 (68.9)	2326
Age (mean ± SD)	62.87 ± 14.09	57.94 ± 15.38	0
Race			
Black	72 (9.9%)	152 (9.5%)	224 (9.6%)
Hispanic	95 (13.1%)	206 (12.9%)	301 (12.9%)
White	481 (66.5%)	1111 (69.3%)	1592 (68.4%)
Education			
Less than ninth grade	33 (4.6%)	41 (2.6%)	74 (3.2%)
Ninth-11th grade	60 (8.3%)	103 (6.4%)	163 (7.0%)
High school graduate/General Educational Development (GED)	161 (22.2%)	288 (18.0%)	449 (19.3%)
Some college or Associate of Arts (AA) degree	193 (26.6%)	573 (35.8%)	766 (32.9%)
College graduate or above	276 (38.1%)	597 (37.3%)	873 (37.5%)
Marital status			
Married	430 (59.4%)	838 (52.3%)	1268 (54.5%)
Not married	292 (40.3%)	764 (47.7%)	1056 (45.4%)
Depression severity			
Minimal (PHQ-9: 0-4)	462 (63.9%)	948 (59.2%)	1410 (60.6%)
Mild (PHQ-9: 5-9)	153 (21.1%)	372 (23.2%)	525 (22.6%)
Moderate (PHQ-9: 10-14)	57 (7.9%)	175 (10.9%)	232 (10.0%)
Moderately severe (15-19)	29 (4.0%)	68 (4.2%)	97 (4.2%)
Severe (PHQ-9: 20-27)	11 (1.5%)	27 (1.7%)	38 (1.6%)

A linear regression analysis was conducted to evaluate whether the presence of LUTS modifies the well-established relationship between gender and depression. Among patients with LUTS, depression severity (measured as a continuous composite PHQ-9 score ranging from 0 to 27) was analyzed while adjusting for age, race, education, and marital status (Table 1). The model showed that gender was not significantly associated with depression severity in this population (β = 0.252, 95% CI: -0.192 to 0.697, p = 0.266). In contrast, age was a highly significant predictor, with each additional year associated with a 0.066-point decrease in depression score (β = -0.066, 95% CI: -0.080 to -0.053, p < 0.001) (Table 2).

**Table 2:** Regression results for depression score among LUTS patients LUTS, lower urinary tract symptom

Variable	Beta	95% CI	p-value
Gender (female vs. male)	0.252	-0.192 to 0.697	0.266
Age (years)	-0.066	-0.080 to -0.053	<0.001
Race: Hispanic (vs. Black)	0.548	-0.291 to 1.387	0.2
Race: White (vs. Black)	0.654	-0.023 to 1.332	0.058
Education: Ninth-11th grade (vs. <9th)	0.039	-1.350 to 1.428	0.956
Education: High school graduate/General Educational Development (GED)	-0.780	-2.041 to 0.480	0.225
Education: Some college or Associate of Arts (AA) degree	-1.153	-2.386 to 0.080	0.067
Education: College graduate or above	-2.267	-3.498 to -1.036	<0.001
Marital status: Not married (vs. married)	1.411	0.999 to 1.823	<0.001

Regarding race, compared to Black patients, Hispanic patients had an estimated 0.55 points higher depression score (β = 0.548, 95% CI: -0.291 to 1.387, p = 0.200), and White patients had an estimated 0.65 points higher score (β = 0.654, 95% CI: -0.023 to 1.332, p = 0.058), though neither of these differences reached statistical significance. In terms of education, patients with a college degree or above had significantly lower depression scores (β = -2.267, 95% CI: -3.498 to -1.036, p < 0.001) compared to those with less than a ninth-grade education, whereas those with some college or an AA degree had lower depression scores (β = -1.153, 95% CI: -2.386 to 0.080, p = 0.067), a difference that was not statistically significant. Finally, not being married was associated with a 1.41 points higher depression score compared to being married (β = 1.411, 95% CI: 0.999 to 1.823, p < 0.001) (Table 2).

## Discussion

The combined demographic profile of the LUTS cohort, with a mean age of 59.47 years and a predominance of females, aligns with existing epidemiological data on urinary dysfunction and its psychosocial consequences [12]. Furthermore, the distribution of depression severity, with a majority of patients exhibiting minimal to mild symptoms, suggests that while LUTS is associated with an increased risk of depression, the majority of patients may not reach clinical thresholds for severe depression.

This study examined the relationship between LUTS and depression in a nationally representative sample from NHANES, with a particular focus on potential gender differences. Our findings indicate that while LUTS and depression are both highly prevalent conditions, the hypothesized gender differences in depression severity among LUTS patients were not observed. Although depression is generally more common among women [9,10], our regression analysis revealed that gender was not a statistically significant predictor of depression severity (β = 0.252, p = 0.266) when controlling for age, race, education, and marital status. This suggests that the presence of LUTS may exert a similar impact on depression scores regardless of gender.

Notably, age emerged as a strong predictor in our model. Each additional year of age was associated with a 0.066 points decrease in the depression score (β = -0.066, p < 0.001), implying that older patients with LUTS tend to report lower levels of depressive symptoms. This counterintuitive finding might reflect a cohort effect or adaptive coping mechanisms developed over time, which merits further exploration.

Educational attainment and marital status also showed significant associations with depression. Patients with a college degree or above had significantly lower depression scores (β = -2.267, p < 0.001) compared to those with less than a ninth-grade education. Additionally, individuals who were not married reported significantly higher depression scores (β = 1.411, p < 0.001) relative to their married counterparts. These results are in line with previous literature that highlights the protective role of higher socioeconomic status and stable social support networks in mental health outcomes [17].

This study's limitations include its cross-sectional design, which precludes causal inferences, and its reliance on self-reported data, which may be subject to reporting biases. Additionally, the NHANES dataset employs top-coding for older ages and may underrepresent certain subgroups. Despite these limitations, our study contributes to the literature by providing a nuanced examination of how demographic factors intersect with LUTS to influence depressive symptoms.

## Conclusions

While depression is generally more common among women, our analysis sought to determine whether LUTS modified this established gender disparity. After adjusting for key demographic factors, we found that gender was not significantly associated with depression severity among individuals with LUTS. This suggests that the presence of LUTS may attenuate gender differences in depression, with other factors such as age, educational attainment, and marital status playing a more substantial role in shaping depressive symptoms within this population. Future research should examine whether specific LUTS subtypes (e.g., urinary incontinence, frequency, urgency) or varying symptom severity differentially affect mental health in men and women.
